# Social isolation as a risk factor for all-cause mortality: Systematic review and meta-analysis of cohort studies

**DOI:** 10.1371/journal.pone.0280308

**Published:** 2023-01-12

**Authors:** Ryo Naito, Martin McKee, Darryl Leong, Shrikant Bangdiwala, Sumathy Rangarajan, Shofiqul Islam, Salim Yusuf

**Affiliations:** 1 Population Health Research Institute, McMaster University, and Hamilton Health Sciences, Hamilton, ON, Canada; 2 Department of Public Health, London School of Hygiene & Tropical Medicine, London, United Kingdom; UCL: University College London, UNITED KINGDOM

## Abstract

**Background:**

Although several epidemiological studies have linked social isolation to increased risk of mortality, the magnitude of any effect is unclear, in part because of the use of different measures of social isolation.

**Objective:**

To examine the association between social isolation and all-cause mortality and investigate whether it differs in various subgroups or populations.

**Data sources:**

We searched for relevant studies in electronic databases: MEDLINE (1946 to December 31, 2021), EMBASE (1974 to December 31, 2021), and PsycINFO (1806 to December 31, 2021).

**Selection criteria:**

We included both prospective and retrospective cohort studies that examined the association between social isolation and all-cause mortality among adults.

**Data collection and analysis:**

Two reviewers screened and extracted data independently. We contacted study authors to obtain missing information whenever possible. Data were pooled using a random effect model to calculate estimates of the effects of social isolation on all-cause mortality.

**Results:**

Data from studies involving 1.30 million individuals were included. The pooled hazard ratio of social isolation for all-cause mortality was 1.33 (95% confidence interval; 1.26–1.41, heterogeneity: Chi² = 112.51, P < 0.00001, I² = 76%).

**Conclusion:**

Social isolation is associated with increased risk for all-cause mortality.

**Registration:**

PROSPERO (CRD42020152351).

## Introduction

Social isolation is characterized as the absence of social relationships [1] that arise from social contacts, social resources, and participation in social or religious activities [2, 3] and has attracted increasing attention as a determinant of poor health [4–9]. Several studies have linked it to an increased risk of all-cause mortality but the magnitude of any effect has varied in these studies with hazard ratio ranging from 1.16 to 3.74 for social isolation. A universal measure of social isolation has not yet been established because of the complex nature of the concept. However, social isolation can be defined with self-reports such as loneliness, and/or objective factors such as minimal contacts or interactions with others, indicating lack of social relationships. Social isolation may influence health-related behaviors through lack of self-interest and loss of motivation. Some people with social isolation might commit suicide as an extreme manifestation of self-destructive behavior [10]. Social isolation could lead to increased morbidity and mortality partly through unhealthy lifestyles which include smoking, excessive alcohol intake, poor nutrition, and physical inactivity [11–13]. Given the increasing isolation experienced by many people during the COVID-19 pandemic, it seems timely to summarize what is known on this topic. In this systematic review and meta-analysis, we examined the available evidence on the association of social isolation with mortality. This information is important for several reasons. At an individual level, it may prompt clinicians and carers to consider social isolation in making a holistic assessment of someone’s risk. At a public policy level, it might influence decisions on investments that strengthen the ability of otherwise isolated people to engage with those around them. However, those seeking to use this information face several problems. First, the existing research has used different approaches and in particular, a variety of measures. Second, this research has been undertaken in differing settings, for example, urban or rural areas or countries at different levels of development, so it is not clear how generalizable different findings are, given differences in social norms, customs, and structures. Third, it is not clear whether the effects vary between men and women. Therefore, we extend previous meta-analyses by including data from a wider range of countries from all regions of the world, at different economic levels and in urban and rural settings. Therefore, our objective in this systematic review is to assess the effects of social isolation on mortality, taking into account variations in different groups—sex, national income, and regions (North America, Europe, and Asia).

## Materials and methods

### Study eligibility criteria

We included data from prospective or retrospective observational cohort studies that reported data on both social isolation and mortality. We included only studies in which social isolation was assessed as an exposure of interest and the participants were followed prospectively for subsequent events. We excluded publications based on case reports case series designs or randomized controlled studies. We applied no exclusion criteria regarding the language of publication, time, or location. Mortality was the outcome of interest and the pooled estimates of its association with social isolation were calculated. Since the effect of social isolation on mortality may vary across included studies due to heterogeneity in study populations, follow-up length, and other factors and sampling variability, random-effects models were used in our meta-analyses.

### Search methods for identification of studies

We searched for studies published before December 2021 using the electronic databases: MEDLINE (1946 to December 31, 2021), EMBASE (1974 to December 31, 2021), and PsycINFO (1806 to December 31, 2021). Details of the search strategy for each database are provided in S1 Appendix. We included thesaurus and free text key terms including social isolation, mortality, death, cohort studies, prospective studies, and retrospective studies. We sought to develop a complex search strategy with the respective Boolean operators and relevant search filters in each database. To complement the electronic database search, we screened reference lists of past reviews and studies meeting the inclusion criteria to maximize the inclusion of potentially relevant studies. Two researchers independently screened titles and abstracts before assessing full records. The full-text screen phase utilized the same approach and duplicate and independent screening and assessment were used in the data abstraction and risk of bias stages. Disagreements were settled by consensus discussion and 3^rd^ party adjudication if needed.

### Data collection and analyses

Two individuals (R.N. and L.S.) independently screened titles and abstracts using Rayyan, an online systematic review tool, to assess potential eligibility for inclusion. The two reviewers then screened full texts to determine whether such trials would be included. After each stage, the reviewers met to resolve disagreements. The PRISMA Checklist is in S2 Appendix. Data were extracted from full texts by one researcher and checked by a second and, any differences were resolved by discussion. Study authors were contacted to obtain missing data.

### Assessment of risk of bias in included studies

The Cochrane Risk Of Bias In Non-randomized Studies—of Interventions (ROBINS-I) [13] tool was used to appraise the quality of included studies. The tool offers a structured and comprehensive approach for the assessment of non-randomized studies. Central features of the ROBINS-I tool include the use of signaling questions to guide the risk of bias judgments within seven bias domains. Quality assessment was carried out by one reviewer and then checked by the other. Any disagreements were discussed and resolved. Where necessary, a third reviewer (P.E.A.) was involved to adjudicate unresolved disagreements. Study authors were contacted for additional information to clarify study methods and to gather missing data.

### Quantitative synthesis

We combined the data from the included studies and calculated the effect size of social isolation on all-cause mortality. The hazard ratio was chosen as a measure of the combined effect size because the majority of the studies reported relative hazards of social isolation and its associated confidence intervals, comparing people with the highest versus the lowest levels of social isolation. Where a group of socially isolated people was used as the reference, results were transformed to the reference group being the one reporting no social isolation in order to allow comparisons across studies. Only papers for which an effect estimate and standard error or confidence interval were available or could be calculated were included in analyses to calculate the pooled estimate. Information on the degree of adjustment for covariates of the estimates in each study is described in the results section. Where a study analyzed multiple models to calculate an effect size, we extracted data from the most adjusted Cox proportional hazard model to minimize the risk of confounding. To address heterogeneity among countries we undertook subgroup analyses, disaggregating data by national income using the World Bank classification (high-, middle-, and low-income countries) and, also by geography (North America, Europe, and Asia). We used RevMan V.5.3 (Review Manager (RevMan) Version 5.3) to calculate effect estimates, build forest and funnel plots, and assess heterogeneity among studies using the I^2^ statistic.

## Results

**Fig 1:**
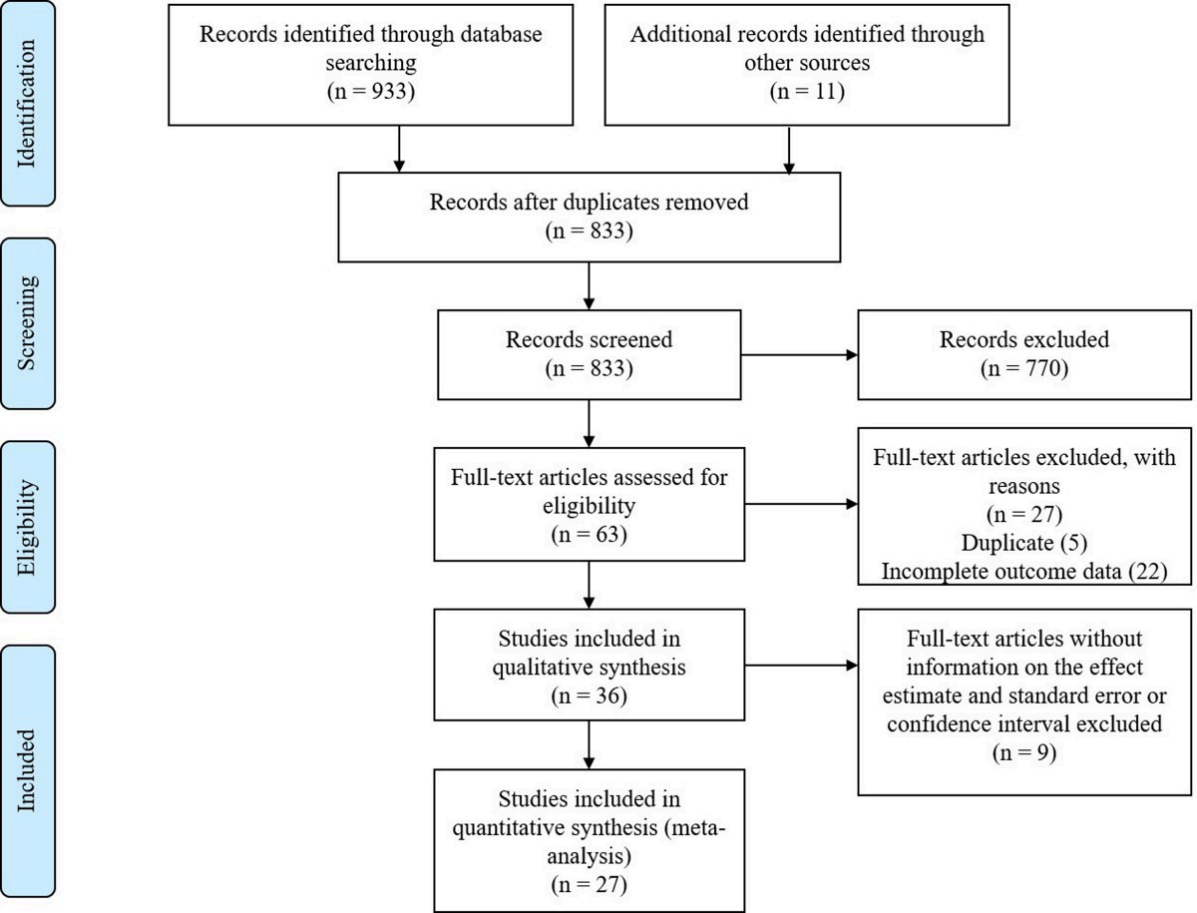
PRISMA flow diagram. A total of 36 studies were identified for inclusion in the reviews, after a two-stage screening process. Twenty-seven studies among them were included in meta-analyses.

Thirty-six studies [2, 4, 6–9, 12, 14–42] were identified for inclusion in this review, after a two-stage screening process. A flow diagram of the study selection process is presented in Fig 1. A summary of the descriptive characteristics of the evidence included in this review is in Table 1. Twenty articles were from North America (one study was conducted in U.S. and China), 12 from Europe (one study was conducted in UK and Japan), and 3 from Asia (Japan; 2, Taiwan; 1), and 1 article (PURE) included an international cohort with participants from 20 countries (Canada, Saudi Arabia, Sweden, the United Arab Emirates, Argentina, Brazil, Chile, Colombia, Iran, Malaysia, Palestine, Philippines, Poland, South Africa, Turkey, Bangladesh, India, Pakistan, Tanzania, and Zimbabwe.). These studies included data on a total of 1,399,572 participants. Overall, the weighted mean age of participants at entry into the study was 53 years. They were followed for an average of 17 years (range from 8 months to 30 years). As noted above, given the wide range of follow-up periods, the Cox regression models was applicable given that the hazard ratios (HR) were constant over time.

**Table 1:** Characteristics of 36 studies included in this systematic review.

Author, year published	Country	Data collection dates	Number of study population	Age of study subjects at baseline	Follow-up period	Adjustment for covariates	Number of events
Alcaraz, 2019	USA	1982–1983	580,182	54.9	30 years	Stratified on single-year of age and race, and adjusted for sex, smoking status, education, body mass index, and history of diabetes.	250,006
Beller, 2018	Germany	1996–2015	4,838	60.09	20.83 years	Age, gender, the number of chronic diseases, marital status, and education	1,079
Berkman, 1979	USA	1965	4,725	47	9 years	Age	371
Berkman, 2004	France	1991	16,699	40–50 (men), 35–50 (women)	8 years	Age, occupational grade, cigarette smoking, alcohol consumption, body mass index, depressive symptoms, self-rated health and geographic region of France	men; 228 women; 29
Brummett, 2001	USA	1992	430	63.6	47.3 months	Age, number of diseased vessels, left ventricular ejection fraction, presence or absence of congestive heart failure, and comorbidity.	159
Cerhan, 1997	USA	1982–1985	2,575	79.1 (men), 80.3 (women)	8 years	Age, education, smoking status, onset of major illness, change in physical function, change in self-perceived health status, change in depressive symptoms, and memory recall test	1,059
Crowe, 2021	USA	2006–2014	11,302	50–95	59.2 months	Age, age-squared, sex, age–sex interactions, race/ethnicity, and a dummy variable coding whether participants were assigned to the subsample of the U.S. Health and Retirement Study	1,096
Elovainio, 2017	UK	2007–2010	466,901	56.5	6.5 years	Age, sex, ethnicity, body mass index, diastolic blood pressure, systolic blood pressure, grip strength, alcohol consumption, physical activity, smoking, education, household income and Townsend Deprivation Index, depressive symptoms and history of chronic illness.	11,593
Eng, 2002	USA	1988	28,369	55.2	10 years	Age, occupation, health behaviors, general physical condition, coronary risk factors, and dietary habits	1,365
Greysen, 2013	USA	2002–2008	1,836	61	2 years	Age, race, comorbidities, income, depression and alcohol abuse	550
Gronewold, 2020	Germany	2000–2018	4,139	59.1	13.4 years	Age, sex, social integration or social support, systolic blood pressure, low-density lipoprotein cholesterol, high-density lipoprotein cholesterol, glycated haemoglobin, body mass index, antihypertensive medication, lipid-lowering medication and antidiabetic medication	530
Jenkinson, 1993	UK, Norway, Sweden, and Denmark	1986–1988	1,376	59	3 years	Age group, previous myocardial infarction, hospital complications, history of diabetes, and history of hypertension	247
Kaplan, 1988	Finland	1972–1977	13,301	43.8	5 years	Age, province, urban/rural, serum cholesterol, mean weighted blood pressure, cigarettes, body mass index, previous diseases, education and cohort	598
Kawachi, 1996	USA	1988	32,624	60	4 years	Age, time period, smoking, hypertension, diabetes, high cholesterol, a history of angina pectoris, myocardial infarction, body mass index, alcohol intake and physical activity	511
Keller, 2003	USA	1988–1999	1,270	78.4	4 years	Age, income, education, Instrumental Activities of Daily Living, the Cumulative Illness Rating Scale, and informal service use	Not available
Kraav, 2021	Finland	1984–2011	2,588	42–61	23.2 years	Age, year of examination, adulthood socioeconomic status, alcohol consumption, smoking, and physical activity, Baltic Sea Diet Score, hours slept at night, Human Population Laboratory depression scores, high-sensitivity C-reactive protein, systolic blood pressure, low-density lipoprotein cholesterol, body mass index, and past history of cardiovascular disease	1,132
Kreibig, 2014	USA	2000–2002	1,019	66.9	6.7 years	Age, ethnicity, body mass index, income, left ventricular ejection fraction, inducible ischemia, chronic obstructive pulmonary disease, use of statins and diuretics, and biological mediators	347
Kroenke, 2006	USA	1992–2002	2,835	65.1	6 years	Age, time between diagnosis and assessment of social networks, cancer stage at diagnosis, chemotherapy, tamoxifen, radiation, estrogen-receptor status, age at menarche, oral contraceptive use, birth index, menopausal status, age at menopause, use of hormone replacement therapy, smoking status, body mass index, physical activity, and protein intake	224
Kroenke, 2013	USA	1997–2000	2,264	58.2	10.8 years	Age, time between social assessment and breast cancer diagnosis, disease severity, treatment, education, ethnicity, reproductive variables, body mass index, physical activity, alcohol intake and smoking status	401
Kroenke, 2017	USA and China	1976–2006	9,267	56.8	10.6 years	Age, time between diagnosis and social network assessment, cohort, education, race, stage, estrogen receptor status, human epidermal growth factor receptor 2 status, parity, menopausal status, and comorbidity	1,521
Lennartsson, 2021	Sweden	2004–2009	1,161	78.4	5 years	Age, gender, education, self-rated health, mobility limitations, psychological distress, and cardiovascular problems (at least one severe problem or three slight problems with chest pain, heart problems, or high blood pressure, or a slight or severe problem with heart attack or stroke)	352
Manemann, 2018	USA	2013–2015	1,681	73.3	8 months	Age, sex, education, marital status and Charlson comorbidity index	59
Naito, 2021	20 countries	2005–2015	118,764	50.3	9 years	Age, sex, education attainment (pre-secondary, secondary or post-secondary education), residence area (rural or urban), country income level, smoking, alcohol use, physical inactivity, diet score, hypertension, diabetes, coronary artery disease, depression, and disabilities	9,487
Pantell, 2013	USA	1988–1994	16,849	47.5	14.1 years	Age, race, education, income level, self-reported health status, clinical risk factor, smoking, obesity, high blood pressure and high cholesterol	Not available
Saito, 2012	Japan	2003	13,310	72.8	4 years	Age, sex, educational attainment, marital status, and history of disease and impairment	1,044
Saito, 2021	Japan, UK	2003	20,437	≥ 65	10 years	Age, sex, self‐rated health, presence of medical treatment, marital status, equivalent income and basic activities of daily living	5,945
Sakurai, 2019	Japan	2008	1,023	72.3	6 years	Age, sex, number of years of education, comorbidities, depression symptoms, subjective health, and residential areas	65
Sarma, 2018	USA	1992–2012	896	69.8	9.5 years	Age at diagnosis, year of diagnosis, race/ethnicity, smoking status, alcohol intake, aspirin use, physical activity, body mass index, cancer stage, cancer grade and cancer site	380
Seeman, 1987	USA	1965	4,174	≥ 38	17 years	Age, race, baseline health status, perceived health, depression and health practices	1,219
Schoenbach, 1986	USA	1967–1969	2,059	52.5	13 years	Age, presence of coronary heart disease, stroke, transient ischemic attack, treated diabetes, systolic blood pressure, cholesterol, smoking, Quetelet index, electrocardiogram abnormalities, social status and leisure time physical activity	419
Smith, 2018	UK	2004/2005	7,731	64	8–9 years	Age, sex, ethnicity, education, occupational class, non-pension wealth, limiting longstanding illness, functional impairment, depressive symptoms, cancer, heart disease, stroke, diabetes, arthritis, chronic lung disease, health behaviors, and cognitive dysfunction	1,261
Steptoe, 2013	UK	2004–2005	6,500	≥ 52	7.25 years	Age, sex, wealth, education, marital status, ethnicity, limiting long-standing illness, mobility impairment, cancer, diabetes, coronary heart disease, chronic lung disease, arthritis, stroke, diagnosed depression and Center for Epidemiologic Studies Depression Scale	918
Stokes, 2021	USA	2004–2016	3,975	56.1	11.8 years	Sex, race, education, household income, smoking status, physical activity, diabetes, hypertension, heart diseases, cancer, functional limitation, self-rated health, personality traits, depression, generalized anxiety disorders, K-6 psychological distress, any drug use, and number of alcohol problems	547
Tanskanen, 2016	Finland	1994	8,650	45.0	17 years	Age, gender, self-reported health, body mass index, frequency of heavy alcohol consumption, exercise or sports, educational level, employment or student status, and household income	1,472
Yang, 2013	USA	1988–2006	6,729	≥ 40	18 years	Age, race-ethnicity, education, family income, smoking, drinking, chronic conditions, physical activity, body mass index and self-rated health	2,774
Yu, 2020	Taiwan	2003–2015	1,267	≥ 65	10 years	Age, sex, education, body mass index, smoking, drinking, physical activity, Charlson comorbidity index, and depressive symptoms	593

### Prevalence of social isolation

Social isolation was measured subjectively or objectively using questionnaires. The study participants were mainly from population-based cohorts. The prevalence of social isolation varied from 0.6% [16] to 35.7% [26]. While this range is wide, it may reflect the diversity of assessment tools and populations. Four assessment tools were used in these 36 studies: the SNI (S3 Appendix) [2] in 11, modified SNI in 14, the Mannheim interview on social support in one [15], the Patient-Reported Outcomes Measurement Information System Social Isolation Short Form 4a v2.0. in one study [9], and 9 different tools designed to capture the availability and/or frequency of contacts for the other studies.

#### Impact of social isolation on all-cause mortality

**Fig 2:**
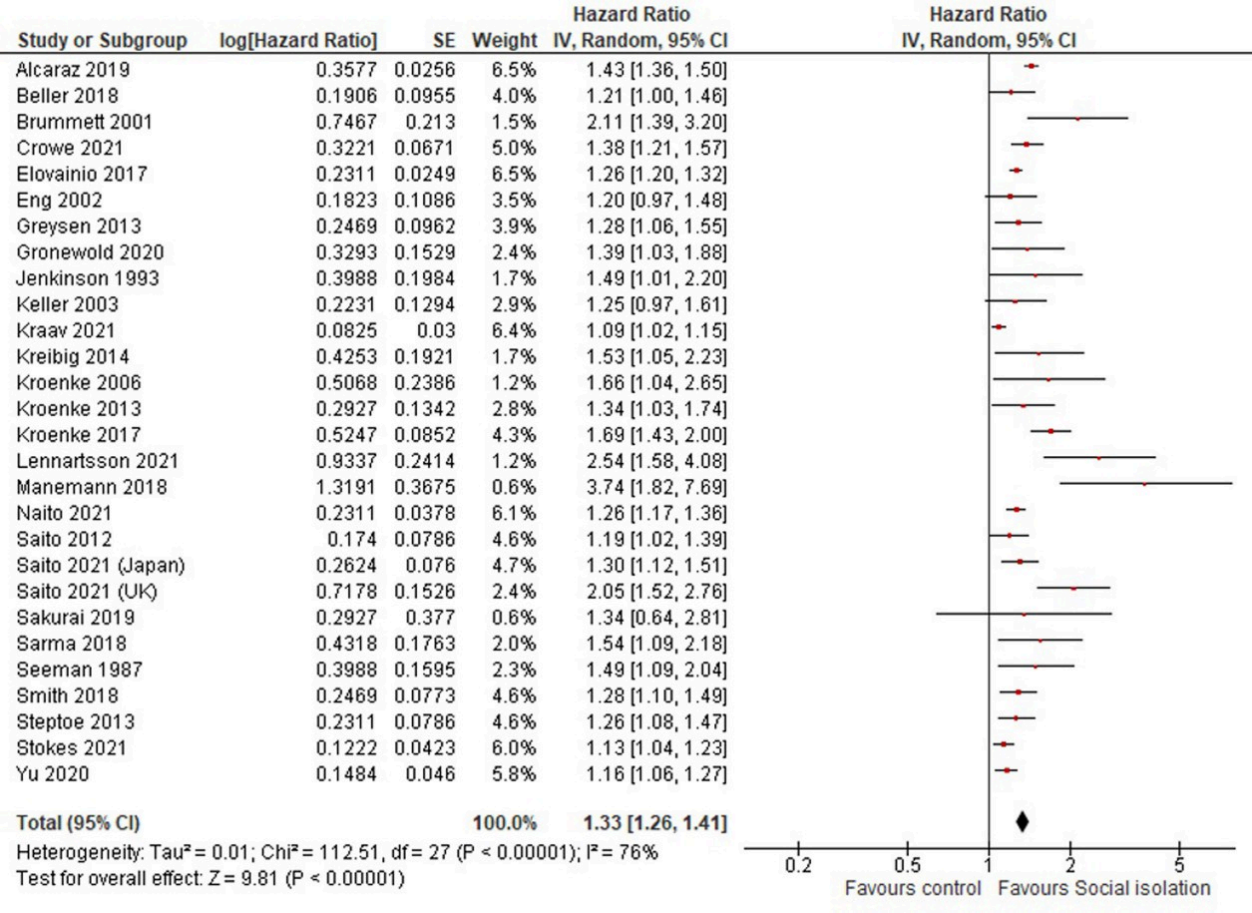
Forest plots and pooled estimates for hazard ratios of social isolation for all-cause mortality. The random effects weighted average hazard ratios of social isolation for all-cause mortality is 1.33 (95% CI); 1.26–1.41) with substantial heterogeneity (heterogeneity: Chi² = 112.51, P < 0.00001, I² = 76%).

**Fig 3:**
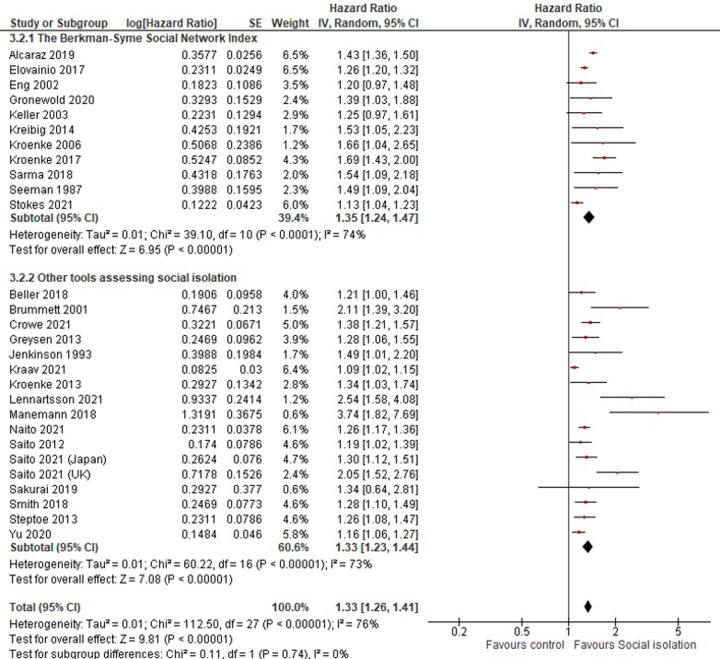
Forest plots and pooled estimates hazard ratios of social isolation for all-cause mortality are shown separately for different social isolation assessment tools. The hazard ratio among the studies using the Berkman-Syme Social Network Index to assess social isolation is 1.35 (95% CI; 1.24–1.47) with substantial heterogeneity (heterogeneity: Chi² = 39.10, P < 0.0001, I² = 74%) whereas that among the remainders is 1.33 (95% CI; 1.23–1.44) with substantial heterogeneity (heterogeneity: Chi² = 60.22, P < 0.00001, I² = 73%).

Twenty-seven [4, 6, 8, 9, 15, 16, 18–20, 23–28, 30–33, 35–42] out of 36 articles for which the effect size and standard error or confidence interval were available or could be calculated were included in meta-analyses to estimate the pooled effects. All of the studies provided HRs adjusted for covariates, which included age, health behaviors (i.e. smoking or alcohol), or comorbidities. The random-effects weighted average HR of social isolation for mortality is 1.33 (95% CI; 1.26–1.41) with substantial heterogeneity (heterogeneity: Chi² = 112.51, P < 0.00001, I² = 76%) (Fig 2). The funnel plot visually indicated publication bias (S4 Appendix), and this was supported by the Egger’s test (p = 0.032). Given the substantial degree of heterogeneity and different type of assessment tools for social isolation used, we conducted subgroup analyses to determine the extent to which the effect estimates were affected by the type of tool used. The SNI was used to assess social isolation in eleven studies [4, 16, 18, 23–25, 27, 32, 33, 39, 42]. The random-effects weighted average HR among these studies is 1.35 (95% CI; 1.24–1.47) with substantial heterogeneity (heterogeneity: Chi² = 39.10, P < 0.0001, I² = 74%) whereas studies using other tools generate a pooled estimate of 1.33 (95% CI; 1.26–1.41) with substantial heterogeneity (heterogeneity: Chi² = 60.22, P < 0.00001, I² = 73%) (Fig 3). The overall HR is strikingly similar irrespective of the tool used to assess social isolation. Subgroups defined by national income of the country yielded similar results, with the values of HR in high-, middle-, and low-income countries of 1.34 (95% CI 1.26–1.42), 1.27 (1.15–1.40), and 1.47 (1.25–1.73), respectively (S5 Appendix). There are, however, differences by region, with the highest HR in North America (HR 1.41, 95% CI 1.28–1.55) followed by Europe (HR 1.33, 95% CI 1.20–1.47) and Asia (HR 1.20, 95% CI 1.12–1.27) (S6 Appendix). This variation might be real, and may be related to the differences in family, community, and social structures. However, it was not possible to explore these possibilities further with the available data.

### Narrative reviews of remaining studies

Narrative reviews included 9 studies that were not included in the meta-analyses because it was not possible to obtain information on the effect estimates of social isolation on all-cause mortality, standard errors, or confidence intervals. Gender-specific effect sizes are provided in 5 studies of which 4 reported statistically significant associations between social isolation and mortality, with HR ranging from 1.50 to 2.70 for men and 1.45 to 3.64 for women [12, 14, 17, 29]. The remaining study showed no significant association between social isolation and all-cause mortality for both genders [34]. Two studies reported inconsistent results [2, 22]. Kaplan et al. reported a significant association between social connections and all-cause mortality with an odds ratio of 1.54 (95% CI; 1.21–1.95) in individuals with low social connections as compared to those with high social connections for men, while no significant association was found for women [21]. In the other study that used a social isolation score with a scale from 0 to 100, this score was associated with mortality in a graded fashion [7].

The risk of bias in the 9 studies was considered moderate. We found inconsistency in the results across the studies and, also the magnitude of the effect sizes varied across the studies. Indirectness was rated “not serious” since the results applied to our research question of this systematic review. Publication bias cannot be eliminated despite extensive attempts to find unpublished studies through clinical study registers. Overall, this narrative synthesis, with 9 studies, is inconsistent with the meta-analysis result showing that social isolation is associated with increased hazard of all-cause mortality.

### Assessment of the risk of bias across studies and the quality of evidence

The Cochrane ROBINS-I was used to appraise the quality of included studies. A summary of the results is in S7 Appendix. All studies except one, are judged to be at moderate risk of bias due to residual confounding because they are non-randomized studies. However, a range of potential confounders at baseline was controlled for. Only one study was judged to be at serious risk of residual confounding, since the multivariate analyses included only one variable other than social isolation status. Bias in the selection of participants is judged to be moderate in all studies as it is plausible, and perhaps inevitable, that some eligible participants who were socially isolated were less likely to participate in the study and to continue to do so over time. Bias due to deviations from intended interventions was judged to be low, moderate, or serious depending on follow-up periods since it is likely that the status of social isolation can be changed overtime if the follow-up period was relatively long. Twenty-eight out of 36 studies are judged to be low risk of bias due to missing data while the other articles are judged to be at moderate risk because they failed to report reasons for missing participants. Bias in the measurement of outcomes was judged to be low in all studies because the outcome of interest was all-cause mortality which has little subjectivity in reporting. Finally, bias in the selection of the reported result is judged to be low among all studies because reported results in each study corresponded to the intended outcome measurement. Based on these assessments, the overall risk of bias across studies is rated as moderate.

## Discussion

### Summary of the main results

This systematic review and meta-analysis found a significant 33% (95% CI; 1.26–1.41) higher hazard of all-cause mortality among those who are socially isolated. The effect sizes are similar using different assessment tools and in studies conducted in different country income levels providing reassurance of the universal importance of social isolation This finding is consistent with the narrative review of studies excluded from the meta-analysis.

Our findings are also consistent with previous reviews [43, 44], as well as another recent meta-analysis, which found an increased likelihood of death of 29% after accounting for multiple covariates [43]. However, our meta-analysis extends the previous one with an additional 7 years of data by including data from a wider range of countries in all parts of the world, at different economic levels and in urban and rural settings. Notably, we can show that the effect sizes observed in different studies of social isolation on mortality are consistent across this extended range. We are also able to show that our findings are robust to the choice of instrument. The similar association was observed for different regions (North America, Europe, and Asia) in the world with relatively low HR in Asia. The narrative review of the 9 that could not be included due to lack of data is inconsistent with the meta-analysis.

### Potential mechanisms linking social isolation and mortality

Previous studies have highlighted pathways through which social relationships can influence mortality. Social isolation is considered to influence health-related behaviors through lack of self-interest and loss of motivation, which originate from lack of social relationships. Individuals with social isolation may adopt unhealthy lifestyles because they give less priority to their health due to their feeling of low self-esteem [45]. In a cohort study, socially isolated men were more likely to be cigarette smokers, heavy drinkers, and to have worse self-rated health, whereas socially isolated women tended to report worse self-rated health and mental health [14]. Previous studies reported that fewer social connections were associated with smoking [46–48]. More social connections were associated with more healthy behaviors such as smoking cessation and physical activity, perhaps because people with social connections might receive advice or support from others or may have sense of obligation to stay healthy for family members and friends. Social isolation is related to excessive alcohol intake and alcohol abuse. People who are socially isolated may consume excessive amounts of alcohol to help them deal with the psychological distress of being isolated [49, 50]. Social isolation is recognized as a risk factor for malnutrition. Research suggests that people with fewer social contacts or living alone have unhealthier diets with lower nutritional quality and less fruit and vegetables [51]. People with social isolation have a higher frequency of binge eating because they may have inadequate self-regulation or they may binge to cope with feelings of loneliness [52]. Physical inactivity has been recognized as an important risk for mortality [53, 54] and morbidity [55]. Previous research has shown that individuals with social isolation are less physically active [11] because they may be less attentive to their health due to low self-esteem [45] or because social isolation is associated with chronic illness and mobility limitations.

It is, however, necessary to consider reverse causality, whereby those with chronic illnesses, such as chronic lung disease, arthritis, and impaired mobility [6], may become socially isolated. People with physical barriers such as disabilities or chronic diseases may be more likely to develop social isolation since their social networks could shrink due to those barriers [56, 57], and psychological barriers including altered mental status and cognitive dysfunction [56]. Therefore, it is possible that people with social isolation are at higher risks of morbidity and mortality than those without because of their background comorbid diseases that could affect their prognoses.

Other potential mechanism explaining the association between social isolation and mortality is accelerating aging due to chronic stress caused by social isolation [58]. The notion is supported by several experimental data that non-handled rats had cognitive impairment and marked hippocampal cell loss compared to handled ones and that isolated rabbits had more extensive atherosclerosis than non-isolated ones [59, 60]. Furthermore, a human study investigating effects of social interaction on cardiovascular response to psychological challenge, subjects without social support had higher systolic and diastolic blood pressure and increased heart rate than those with social support, indicating the association between social relationships and cardiovascular risk [61].

Our narrative review found increased risks in men and women, although one study found a higher risk among women [2] and a Japanese prospective study only found it in women [30]. However, another study reached the opposite conclusion [21]. Interpreting these findings is, however, complex and, maybe contextually bounded. In many societies, men are more likely to adopt unhealthy behaviors such as smoking and heavy episodic drinking, so it is plausible that they may be more likely to exhibit these behaviors if isolated. On the other hand, in some countries, women may have limited social contacts through less economic opportunities in terms of education and occupation due to gender inequalities, which could limit appropriate medical access when needed.

### Applicability of evidence

In this review, most studies from which data could be extracted were from the US, Europe, or Japan, which limits generalizability. However, one large study (PURE) included data from both urban and rural areas in 20 countries from 5 continents and at differing economic levels and reported consistent results which indicates that our findings are generalizable [28]. The studies used different instruments (11 used the SNI) and, although the results were consistent, ideally it would be possible to use the same ones.

### Potential biases in the review process

We attempted to reduce bias in the review process to a minimum by extensively searching available databases and not limiting the search by language. We also ensured that study identification and inclusion, data extraction were carried out by two independent review authors. Publication bias was detected by the funnel plot asymmetry with the Egger’s test. We were unable to obtain missing information from the authors we contacted.

### Implications for clinical practice

Social isolation is a marker of many risk factors, such as socioeconomic adversity, unhealthy lifestyles, lowered mental well-being, and insufficient access to healthcare facilities, all of which could contribute to increased mortality and morbidity. Social isolation might alter adherence to medical treatments through lack of social support [62–64]. With population aging seen worldwide, the impact of social isolation on mortality would be expected to be greater as the number of people with social isolation is projected to increase as they get older, which results in higher demand on taking measures for those vulnerable populations. The British Geriatrics Society and the Royal College of Psychiatrists Faculty of Old Age Psychiatry have recommended that the negative impact of social isolation on health and well-being through increased risk of developing coronary heart disease, stroke, hypertension, dementia, depression, and suicidal thoughts due to social isolation and loneliness should be considered by clinicians. (Position statement on loneliness and social isolation. Available at https://www.rcpsych.ac.uk/docs/default-source/improving-care/better-mh-policy/position-statements/ps06_2019.pdf?sfvrsn=faba759a_2).

### Implications for policy

These findings point to a need for a policy to recognize and act on the risks associated with social isolation. Some examples can be drawn on, such as measures to create and maintain supportive social networks advocated in the U.K., including improving public transport and creating settings that encourage social interactions (Available at https://assets.publishing.service.gov.uk/government/uploads/system/uploads/attachment_data/file/461120/3a_Social_isolation-Full-revised.pdf). In Japan, policies have focused primarily on older people and include promoting ’worry-free living environments’, community-based activities, and easier access to cultural facilities such as libraries where health-promoting activities can take place (Available at https://www.jlgc.org.uk/jp/wp-content/uploads/2014/06/Social-Isolation-and-Local-Government-in-Japan.pdf). More generally, there is a need to address the greater risk experienced by those socially isolated through modifiable risk factors and access to essential services, including health care.

### Limitations

Our review has a few potential limitations. In common with other systematic reviews of observational studies, proof of a causal association between social isolation to mortality cannot be established from observational data, despite the existing evidence on potential mechanisms explaining the association. Although the effect estimates were calculated using HR of each study, which took into account potential confounding factors, we cannot exclude further confounding from unmeasured confounders such as mental illness, personality disorders, or fear of crime, all of which could expose people risk of being socially isolated. Heterogeneity in study population of the included studies may also cause the observed estimates with substantial heterogeneity. Other limitation is selection bias due to HR as the measurement of the effect estimates in this study. Furthermore, HR may change over time during the follow-up period, which makes the observed effects estimates less reliable.

### Future perspectives

Our study suggests that addressing social isolation and providing health care for those with social isolation may reduce the risk for all-cause mortality, but definitive proof requires evaluation in large randomized trials. Such trials would need appropriate study designs such as a cluster randomized controlled trials, given how interventions are likely to be at the level of the community rather than the individual. Future studies should include social isolation at least as a covariate in relation to health consequences.

## Conclusions

This systematic review and meta-analysis show social isolation is associated with increased risk for all-cause mortality among adults. Our study suggests that addressing social isolation and providing health care for those with social isolation may reduce the risk for all-cause mortality.

## Supporting information

S1 Appendix Search strategy for this systematic review. (DOCX)

S2 Appendix PRISMA checklist. (DOCX)

S3 Appendix The Berkman Syme social network index. (DOCX)

S4 Appendix A funnel plot for the hazard ratios of social isolation on all-cause mortality. (DOCX)

S5 Appendix Forest plots and pooled estimates hazard ratios of social isolation for all-cause mortality are shown separately for different country income levels. (DOCX)

S6 Appendix Forest plots and pooled estimates hazard ratios of social isolation for all-cause mortality are shown separately for different regions in the world. (DOCX)

S7 Appendix Results of risk of bias across the studies. (DOCX)
