# Supplementary material for: Social isolation as a risk factor for all-cause mortality: Systematic review and meta-analysis of cohort studies
Source: PLoS One. 2023 Jan 12;18(1):e0280308. doi: 10.1371/journal.pone.0280308 (PMC9836313; doi:10.1371/journal.pone.0280308)

Appendix 1. Search strategy for this systematic review

**Study eligibility criteria**

**Type of study**

In this systematic review, we included studies that provide quantitative data regarding individuals’ mortality and status of social isolation. We included only studies that are prospective or retrospective cohorts in which social isolation was assessed in study participants at the study entry. Eligible studies would have examined and reported the incidence of mortality over the follow-up period. We excluded publications that are a case-report or case-series design. We applied no exclusion criteria regarding language in publication, or time or location.

**Type of participants**

We included studies in which adults aged 18 years or older were involved and the effect of social isolation on all-cause mortality were evaluated among them.

**Type of exposure**

People with social isolation measured subjectively or objectively using assessment scales were compared to those without social isolation.

**Outcome measures**

All-cause mortality was examined as the outcome and the pooled estimates of social isolation as a risk of all-cause mortality were calculated.

**Search methods for identification of studies**

We searched for studies published up until December 2021 using electronic databases: MEDLINE (1946 to December 2021), EMBASE (1974 to December 2021), and PsycINFO (1806 to December 2021). We included a thesaurus and free text key terms including social isolation, mortality, death, cohort studies, prospective studies, and retrospective studies. We sought expert medical librarian support when needed and ensure that a complex search strategy is used with the respective Boolean operators and relevant search filters in each database. To complement the electronic database search, we screened reference lists of past reviews and studies meeting the inclusion criteria. This was to ensure that potentially relevant studies are considered in our screening process. Two researchers independently and in duplicate, screened titles and abstracts before assessing full records. The full-text screen phase utilized the same approach. Moreover, the data abstraction and risk of bias stages of this review also utilized duplicate and independent screening and assessment. Disagreements were settled by consensus discussion and 3^rd^ party adjudication were used if needed.

**Searching other resources**

We examined the reference lists of eligible articles to find other relevant studies.

**Full electronic search strategies**

1. MEDLINE search strategy

Database: OVID Medline Epub Ahead of Print, In-Process & Other Non-Indexed Citations, Ovid MEDLINE(R) Daily and Ovid MEDLINE(R) 1946 to Present

Searched online 12/31/2021 Strategy saved as social isolation MEDLINE


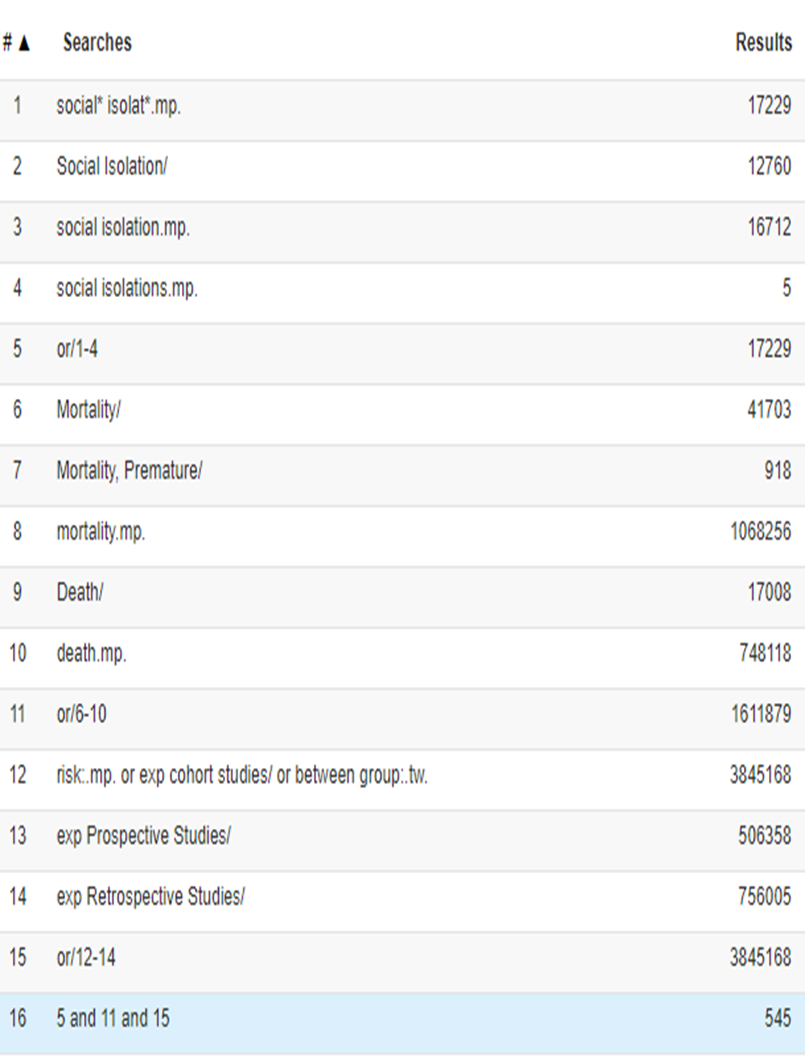


2. Embase search strategy

Database: Embase 1974 to Present

Searched online 12/31/21 Strategy saved as: social isolation Embase
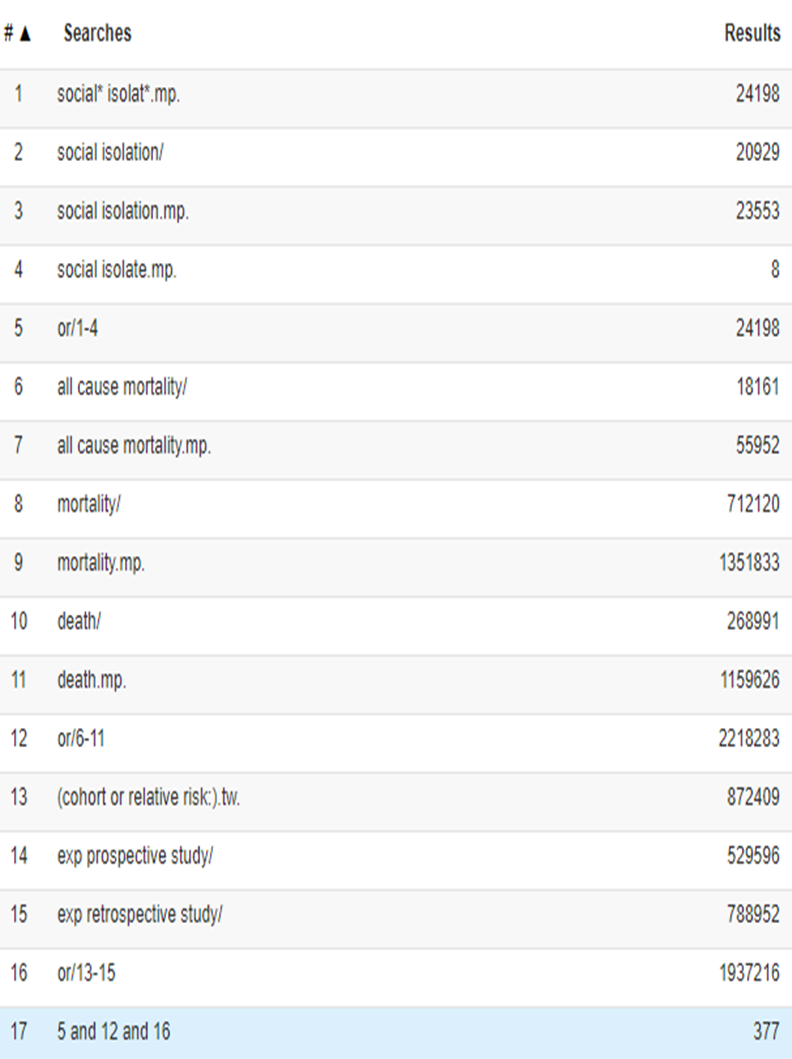


3. PsycINFO search strategy

Database: PsycINFO 1806 to present

Searched online 12/31/21 Strategy saved as social isolation PsycINFO


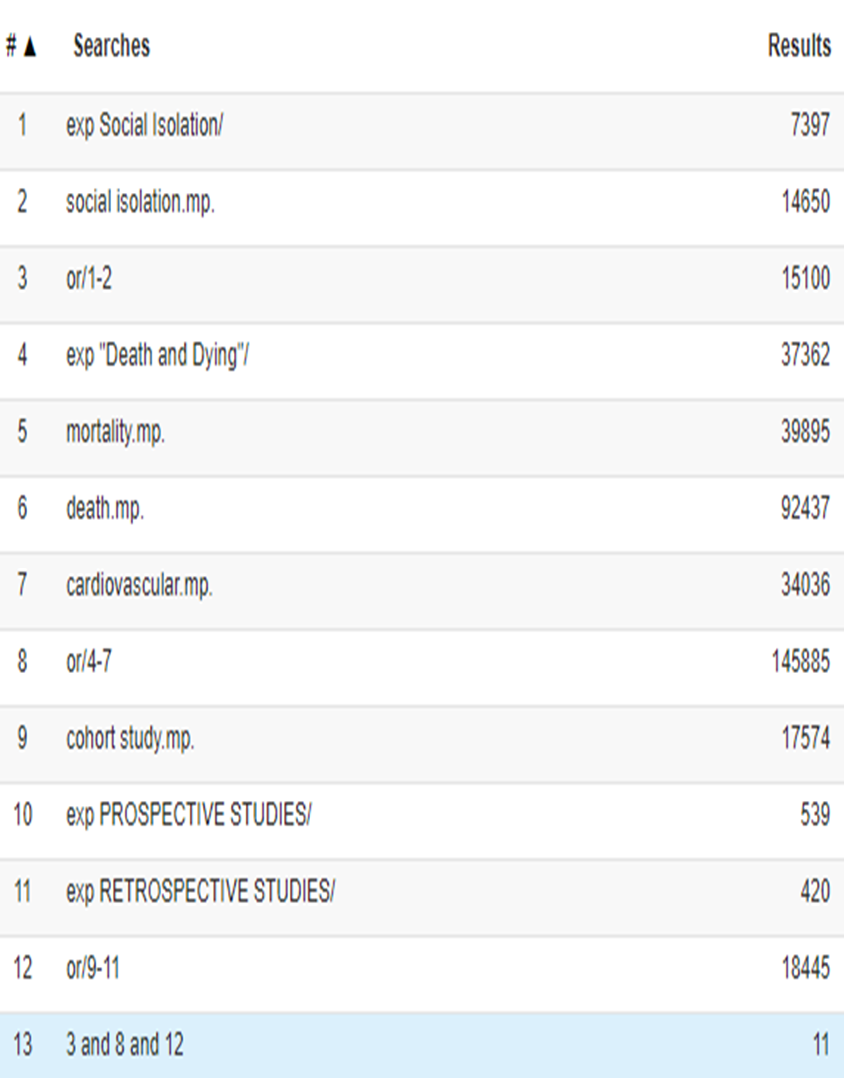

Supplement: S1 Appendix — (DOCX) [file pone.0280308.s001.docx]
