# Supplementary material for: Social isolation as a risk factor for all-cause mortality: Systematic review and meta-analysis of cohort studies
Source: PLoS One. 2023 Jan 12;18(1):e0280308. doi: 10.1371/journal.pone.0280308 (PMC9836313; doi:10.1371/journal.pone.0280308)
Supplement: S3 Appendix — (DOCX) [file pone.0280308.s003.docx]

Appendix 3. The Berkman-Syme Social Network Index

| Protocol Text | The following two-page questionnaire asks about your social support. Please read the following questions and circle the response that most closely describes your current situation.  1. How many *close friends* do you have, people that you feel at ease with, can talk to about private matters?  0: None, 1: 1 or 2, 2: 3 to 5, 3: 6 to 9, 4: 10 or more, 9: Unknown  2. How many of these *close friends* do you see at least once a month?  0: None, 1: 1 or 2, 2: 3 to 5, 3: 6 to 9, 4: 10 or more, 9: Unknown  3. How many *relatives* do you have, people that you feel at ease with, can talk to about private matters?  0: None, 1: 1 or 2, 2: 3 to 5, 3: 6 to 9, 4: 10 or more, 9: Unknown  4. How many of these *relatives* do you see at least once a month?  0: None, 1: 1 or 2, 2: 3 to 5, 3: 6 to 9, 4: 10 or more, 9: Unknown  5. Do you participate in any groups, such as a senior center, social or work group, religious-connected group, self-help group, or charity, public service, or community group?  0: No, 1: Yes, 9: Unknown  6. About how often do you go to religious meetings or services?  0: Never or almost never, 1: Once or twice a year, 2: Every few months, 3: Once or twice a month, 4: Once a week, 5: More than once a week, 9: Unknown  7. Is there someone available to you whom you can count on to listen to you when you need to talk?  0: None, 1: 1 or 2, 2: 3 to 5, 3: 6 to 9, 4: 10 or more, 9: Unknown  8. Is there someone available to give you good advice about a problem?  0: None, 1: 1 or 2, 2: 3 to 5, 3: 6 to 9, 4: 10 or more, 9: Unknown  9. Is there someone available to you who shows you love and affection?  0: None, 1: 1 or 2, 2: 3 to 5, 3: 6 to 9, 4: 10 or more, 9: Unknown  10. Can you count on anyone to provide you with emotional support (talking over problems or helping you make a difficult decision)?  0: None, 1: 1 or 2, 2: 3 to 5, 3: 6 to 9, 4: 10 or more, 9: Unknown  11. Do you have as much contact as you would like with someone you feel close to, someone in whom you can trust and confide?  0: None, 1: 1 or 2, 2: 3 to 5, 3: 6 to 9, 4: 10 or more, 9: Unknown  Scoring Instructions:  Married (no = 0; yes = 1); close friends and relatives (0–2 friends and 0–2 relatives = 0; all other scores = 1); group participation (no = 0; yes = 1); participation in religious meetings or services (less than or equal to every few months = 0; greater than or equal to once or twice a month = 1). The latter two categories were mutually exclusive from each other. Scores were summed: 0 or 1 being the most isolated category; and 2, 3, or 4 formed the other three categories of increasing social connectedness.  Psychometrics on the Berkman-Syme Social Network Index (SNI) and additional evidence for the scale’s predictive validity are available in Berkman and Breslow (1983). |
| --- | --- |
| References | Berkman, L. F., Blumenthal, J., Burg, M., [Carney, R. M](http://www.ncbi.nlm.nih.gov/pubmed?term=%22Carney%20RM%22%5BAuthor%5D)., [Catellier, D](http://www.ncbi.nlm.nih.gov/pubmed?term=%22Catellier%20D%22%5BAuthor%5D)., [Cowan, M. J](http://www.ncbi.nlm.nih.gov/pubmed?term=%22Cowan%20MJ%22%5BAuthor%5D)., [Czajkowski, S. M](http://www.ncbi.nlm.nih.gov/pubmed?term=%22Czajkowski%20SM%22%5BAuthor%5D)., [DeBusk, R](http://www.ncbi.nlm.nih.gov/pubmed?term=%22DeBusk%20R%22%5BAuthor%5D)., [Hosking, J](http://www.ncbi.nlm.nih.gov/pubmed?term=%22Hosking%20J%22%5BAuthor%5D)., [Jaffe, A](http://www.ncbi.nlm.nih.gov/pubmed?term=%22Jaffe%20A%22%5BAuthor%5D)., [Kaufmann, P. G](http://www.ncbi.nlm.nih.gov/pubmed?term=%22Kaufmann%20PG%22%5BAuthor%5D)., [Mitchell, P](http://www.ncbi.nlm.nih.gov/pubmed?term=%22Mitchell%20P%22%5BAuthor%5D)., [Norman, J](http://www.ncbi.nlm.nih.gov/pubmed?term=%22Norman%20J%22%5BAuthor%5D)., [Powell, L. H](http://www.ncbi.nlm.nih.gov/pubmed?term=%22Powell%20LH%22%5BAuthor%5D)., [Raczynski, J. M](http://www.ncbi.nlm.nih.gov/pubmed?term=%22Raczynski%20JM%22%5BAuthor%5D)., & [Schneiderman, N](http://www.ncbi.nlm.nih.gov/pubmed?term=%22Schneiderman%20N%22%5BAuthor%5D).; [Enhancing Recovery in Coronary Heart Disease Patients Investigators (ENRICHD)](http://www.ncbi.nlm.nih.gov/pubmed?term=%22Enhancing%20Recovery%20in%20Coronary%20Heart%20Disease%20Patients%20Investigators%20(ENRICHD)%22%5BCorporate%20Author%5D). (2003). Effects of treating depression and low-perceived social support on clinical events after myocardial infarction: The Enhancing Recovery in Coronary Heart Disease Patients (ENRICHD) randomized trial. *Journal of the American Medical Association, 289*(23), 2106–3116.  Berkman, L., F., & Breslow, L. (1983). *Health and ways of living.* New York: Oxford University Press.  Berkman, L. F., & Syme, S. L. (1979). Social networks, host resistance, and mortality: A nine-year follow-up of Alameda county residents. *American Journal of Epidemiology, 109,* 186–204. |
