# Supplementary material for: Social isolation as a risk factor for all-cause mortality: Systematic review and meta-analysis of cohort studies
Source: PLoS One. 2023 Jan 12;18(1):e0280308. doi: 10.1371/journal.pone.0280308 (PMC9836313; doi:10.1371/journal.pone.0280308)
Supplement: S4 Appendix — (DOCX) [file pone.0280308.s004.docx]

Appendix 4. A funnel plot for the hazard ratios of social isolation on all-cause mortality

Legend. Significant asymmetry is found in the plots for the hazard ratios of social isolation for all-cause mortality


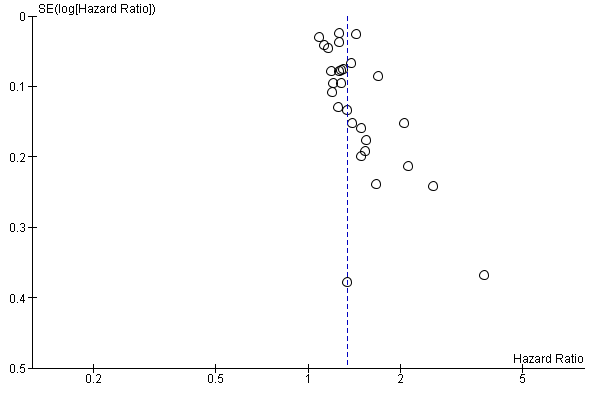


Egger’s test

p = 0.032
