# Supplementary material for: Social isolation as a risk factor for all-cause mortality: Systematic review and meta-analysis of cohort studies
Source: PLoS One. 2023 Jan 12;18(1):e0280308. doi: 10.1371/journal.pone.0280308 (PMC9836313; doi:10.1371/journal.pone.0280308)

Appendix 5. Forest plots and pooled estimates hazard ratios of social isolation for all-cause mortality are shown separately for different country income levels

Legend. The hazard ratio of social isolation for all-cause mortality is consistently high in high-, middle-, and low-income countries.


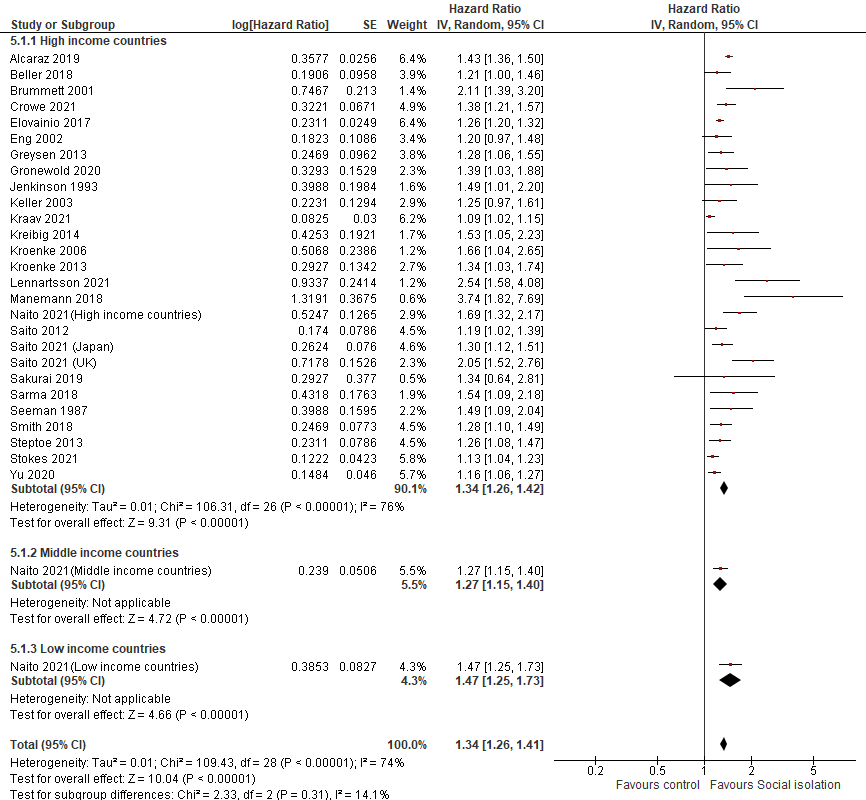

Supplement: S5 Appendix — (DOCX) [file pone.0280308.s005.docx]
