# Supplementary material for: Social isolation as a risk factor for all-cause mortality: Systematic review and meta-analysis of cohort studies
Source: PLoS One. 2023 Jan 12;18(1):e0280308. doi: 10.1371/journal.pone.0280308 (PMC9836313; doi:10.1371/journal.pone.0280308)

Appendix 6. Forest plots and pooled estimates hazard ratios of social isolation for all-cause mortality are shown separately for different regions in the world

Legend. The hazard ratio of social isolation for all-cause mortality is highest in North America followed by Europe and Asia.


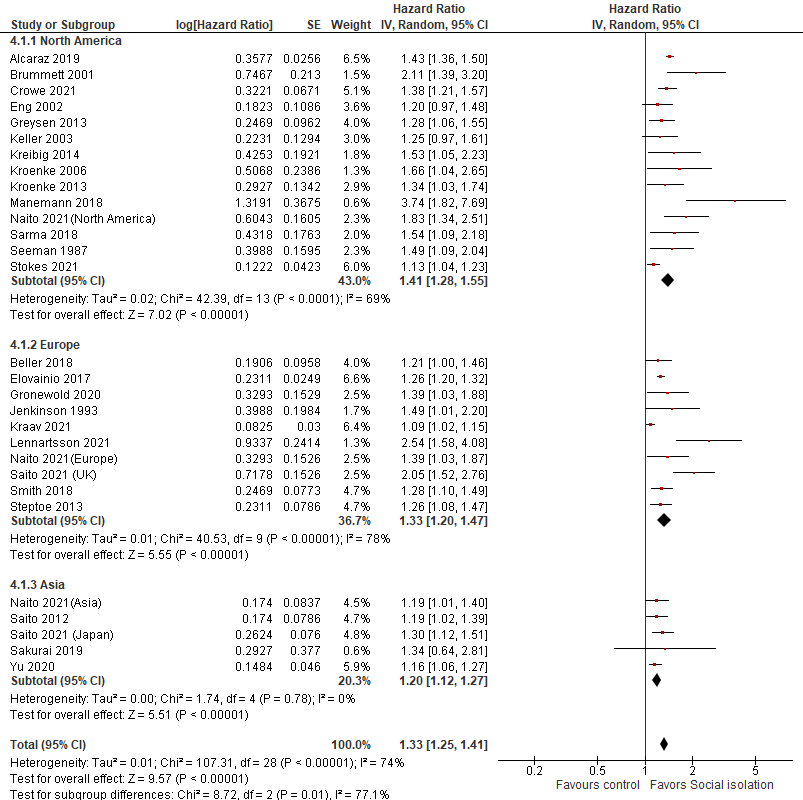

Supplement: S6 Appendix — (DOCX) [file pone.0280308.s006.docx]
