# Supplementary material for: Social isolation as a risk factor for all-cause mortality: Systematic review and meta-analysis of cohort studies
Source: PLoS One. 2023 Jan 12;18(1):e0280308. doi: 10.1371/journal.pone.0280308 (PMC9836313; doi:10.1371/journal.pone.0280308)
Supplement: S7 Appendix — (DOCX) [file pone.0280308.s007.docx]

Appendix 7. Results of risk of bias across the studies

| First author & year published | Bias due to confounding | Bias in selection of participants into the study | Bias in classification of interventions | Bias due to deviations from intended intervention | Bias due to missing data | Bias in measurement of outcomes | Bias in selection of  reported result | Overall |
| --- | --- | --- | --- | --- | --- | --- | --- | --- |
| Alcaraz, 2019 | Moderate risk | Moderate risk | Low risk | Serious risk | Low risk | Low risk | Low risk | Serious risk |
| Beller, 2018 | Moderate risk | Moderate risk | Low risk | Serious risk | Low risk | Low risk | Low risk | Serious risk |
| Berkman, 1979 | Serious risk | Moderate risk | Low risk | Moderate risk | Low risk | Low risk | Low risk | Serious risk |
| Berkman, 2004 | Moderate risk | Moderate risk | Low risk | Moderate risk | Moderate risk | Low risk | Low risk | Low or moderate risk |
| Brummett, 2001 | Moderate risk | Moderate risk | Low risk | Low risk | Moderate risk | Low risk | Low risk | Low or moderate risk |
| Cerhan, 1997 | Moderate risk | Moderate risk | Low risk | Moderate risk | Moderate risk | Low risk | Low risk | Low or moderate risk |
| Crowe, 2021 | Moderate risk | Moderate risk | Low risk | Low risk | Low risk | Low risk | Low risk | Low or moderate risk |
| Elovainio, 2017 | Moderate risk | Moderate risk | Low risk | Moderate risk | Low risk | Low risk | Low risk | Low or moderate risk |
| Eng, 2002 | Moderate risk | Moderate risk | Low risk | Moderate risk | Moderate risk | Low risk | Low risk | Low or moderate risk |
| Greysen, 2013 | Moderate risk | Moderate risk | Low risk | Low risk | Low risk | Low risk | Low risk | Low or moderate risk |
| Gronewold, 2020 | Moderate risk | Moderate risk | Low risk | Serious risk | Low risk | Low risk | Low risk | Serious risk |
| Jenkinson, 1993 | Moderate risk | Moderate risk | Low risk | Low risk | Moderate risk | Low risk | Low risk | Low or moderate risk |
| Kaplan, 1988 | Moderate risk | Moderate risk | Low risk | Moderate risk | Low risk | Low risk | Low risk | Low or moderate risk |
| Kawachi, 1996 | Moderate risk | Moderate risk | Low risk | Low risk | Low risk | Low risk | Low risk | Low or moderate risk |
| Keller, 2003 | Moderate risk | Moderate risk | Low risk | Low risk | Low risk | Low risk | Low risk | Low or moderate risk |
| Kraav, 2021 | Moderate risk | Moderate risk | Low risk | Serious risk | Low risk | Low risk | Low risk | Serious risk |
| Kreibig, 2014 | Moderate risk | Moderate risk | Low risk | Moderate risk | Low risk | Low risk | Low risk | Low or moderate risk |
| Kroenke, 2006 | Moderate risk | Moderate risk | Low risk | Moderate risk | Low risk | Low risk | Low risk | Low or moderate risk |
| Kroenke, 2013 | Moderate risk | Moderate risk | Low risk | Serious risk | Low risk | Low risk | Low risk | Serious risk |
| Kroenke, 2017 | Moderate risk | Moderate risk | Low risk | Serious risk | Low risk | Low risk | Low risk | Serious risk |
| Lennartsson, 2021 | Moderate risk | Moderate risk | Low risk | Moderate risk | Low risk | Low risk | Low risk | Low or moderate risk |
| Manemann, 2018 | Moderate risk | Moderate risk | Low risk | Low risk | Low risk | Low risk | Low risk | Low or moderate risk |
| Naito, 2021 | Moderate risk | Moderate risk | Low risk | Moderate risk | Low risk | Low risk | Low risk | Low or moderate risk |
| Pantell, 2013 | Moderate risk | Moderate risk | Low risk | Serious risk | Low risk | Low risk | Low risk | Serious risk |
| Saito, 2012 | Moderate risk | Moderate risk | Low risk | Low risk | Low risk | Low risk | Low risk | Low or moderate risk |
| Saito, 2021 | Moderate risk | Moderate risk | Low risk | Moderate risk | Low risk | Low risk | Low risk | Low or moderate risk |
| Sakurai, 2019 | Moderate risk | Moderate risk | Low risk | Moderate risk | Moderate risk | Low risk | Low risk | Low or moderate risk |
| Sarma, 2018 | Moderate risk | Moderate risk | Low risk | Moderate risk | Low risk | Low risk | Low risk | Low or moderate risk |
| Seeman, 1987 | Moderate risk | Moderate risk | Low risk | Serious risk | Low risk | Low risk | Low risk | Serious risk |
| Schoenbach, 1986 | Moderate risk | Moderate risk | Low risk | Serious risk | Moderate risk | Low risk | Low risk | Serious risk |
| Smith, 2018 | Moderate risk | Moderate risk | Low risk | Moderate risk | Low risk | Low risk | Low risk | Low or moderate risk |
| Steptoe, 2013 | Moderate risk | Moderate risk | Low risk | Moderate risk | Low risk | Low risk | Low risk | Low or moderate risk |
| Stokes, 2021 | Moderate risk | Moderate risk | Low risk | Serious risk | Low risk | Low risk | Low risk | Serious risk |
| Tanskanen , 2016 | Moderate risk | Moderate risk | Low risk | Serious risk | Low risk | Low risk | Low risk | Serious risk |
| Yang, 2013 | Moderate risk | Moderate risk | Low risk | Serious risk | Low risk | Low risk | Low risk | Serious risk |
| Yu, 2020 | Moderate risk | Moderate risk | Low risk | Moderate risk | Moderate risk | Low risk | Low risk | Low or moderate risk |
